# Single Molecule Fluorescence Microscopy and Machine Learning for Rhesus D Antigen Classification

**DOI:** 10.1038/srep32317

**Published:** 2016-09-01

**Authors:** Daniela M. Borgmann, Sandra Mayr, Helene Polin, Susanne Schaller, Viktoria Dorfer, Lisa Obritzberger, Tanja Endmayr, Christian Gabriel, Stephan M. Winkler, Jaroslaw Jacak

**Affiliations:** 1 University of Applied Sciences Upper Austria, School of Informatics, Communications and Media, Softwarepark 11, 4232 Hagenberg, Austria; 2 University of Applied Sciences Upper Austria, School of Applied Health and Social Sciences, Garnisonstrasse 21, 4020 Linz, Austria; 3 Red Cross Transfusion Service for Upper Austria, Krankenhausstrasse 7, 4020 Linz, Austria

## Abstract

In transfusion medicine, the identification of the Rhesus D type is important to prevent anti-D immunisation in Rhesus D negative recipients. In particular, the detection of the very low expressed DEL phenotype is crucial and hence constitutes the bottleneck of standard immunohaematology. The current method of choice, adsorption-elution, does not provide unambiguous results. We have developed a complementary method of high sensitivity that allows reliable identification of D antigen expression. Here, we present a workflow composed of high-resolution fluorescence microscopy, image processing, and machine learning that - for the first time - enables the identification of even small amounts of D antigen on the cellular level. The high sensitivity of our technique captures the full range of D antigen expression (including D+, weak D, DEL, D−), allows automated population analyses, and results in classification test accuracies of up to 96%, even for very low expressed phenotypes.

The high immunogenicity of the Rhesus factor^1^ renders it one of the most relevant blood markers in transfusion medicine next to the factors of the AB0 system^2^. The Rhesus antigens are encoded by two homologous genes, *RHCE* and the clinically more relevant *RHD*^3^. Currently, more than 280 *RHD* alleles^4^ and approximately 30 Rhesus D (RhD) epitopes are known and account for the strong immunogenicity and the huge complexity of the RhD blood group assignment. Accurate classification of blood samples, however, is of utmost importance as a false assignment can cause dangerous anti-D immunisations potentially leading to haemolytic transfusion incidents or maternal alloimmunisation inducing haemolytic disease of the newborn^5^,^6^.

The *RHD* gene encodes the Rhesus D protein expressed on erythrocyte membranes (Supplementary Fig. 1). Based on the molecular background, the D antigen expression level, and the presence of epitopes, the following five types are defined^7^,^8^ (Table 1): D-positive (D+; high expression), D-negative (D−; no expression due to gene deletion), partial D (extracellular mutations that do not affect the expression level but the existence of certain epitopes), weak D (intracellular or transmembrane mutations causing reduced expression), and DEL (D-elute; intracellular or transmembrane mutations leading to very low expression). The DEL variant^9^ primarily occurs in Eastern Asia (up to 10–30% of all seemingly D− typed individuals^10^,^11^). DEL phenotypes are caused by different *RHD* missense mutations, splice site mutations, or *RHD-CE* hybrid genes^12^,^13^. To date, 32 DEL types are listed in the RhesusBase Site and unidentified ones are still emerging^14^,^15^,^16^ (http://www.rhesusbase.info^17^).

Rhesus D phenotyping of blood donors is routinely performed by incubation of blood samples with anti-D antibodies and visual observation of haemagglutination. This method, however, does not identify all D phenotypes due to a lack of sensitivity. Therefore, very low expressed D variants are frequently misclassified as D--15,18,19. Numerous studies report primary and secondary anti-D immunisation of D− recipients after transfusion of weak D and DEL products^12^,^19^,^20^. Recently, population surveys, lookbacks, and multi-centre studies were performed worldwide revealing the presence of weak D and DEL samples in the seemingly D− donor pool by performing PCR and sequencing. Thus, 0.1% of Caucasians and almost half of Asians (46%) with D− phenotype were reclassified as DEL^6^,^11^,^15^,^21^. While more precise D− characterisation constitutes a loophole in the legislation of most countries, regulations that dictate a more detailed analysis of D− donors were recently introduced in the United States^18^ and Switzerland^22^. *RHD* genotyping of D− samples is performed by molecular biology. Information about protein abundance of known alleles is given by the RhesusBase. In case of novel alleles the D antigen expression level has to be quantified, commonly by flow cytometry^23^: Using flow cytometry, the D antigen is detected by fluorescently labelled standard antibodies^24^,^25^,^26^. This technique, however, exhibits huge inter-laboratory variability^27^, lacks standardised reagents^28^, and frequently fails to detect D variants with low expression due to an inherent lack of sensitivity (limit of detection: 22 antigens/cell)^13^,^14^,^19^. Identification of very low expressed D variants is commonly performed by adsorption-elution assay^9^,^26^,^29^,^30^,^31^,^32^. This laborious technique, however, requires experience, is time-consuming, and lacks standardised protocols. Varying numbers of washing steps and/or different incubation times may lead to contradictory results. DEL samples yielding inconsistent results, as well as DEL samples without detectable D antigen expression, were reported in the literature^3^,^14^,^15^,^33^.

We present a method that allows reproducible classification of all known D types in particular potentially immunogenic, very low expressed D variants. The workflow combines high-resolution fluorescence microscopy and bioinformatic algorithms. The D antigen is labelled with three fluorescent antibodies that are also used in standard immunohaematology. Sequentially, three replicas of each specimen are marked with antibodies binding to different D antigen epitopes. Samples are analysed by high-resolution fluorescence microscopy allowing single molecule sensitive detection. The high sensitivity of our technique captures the D antigen expression of D+, weak D, DEL, and D− phenotypes. Protein expression levels are observed directly at single cell level and reveal huge variations within and between each population. These variations in RhD expression are caused by the stochastic nature of protein expression attributable to the statistical behaviour of chemical systems^34^,^35^,^36^. Image processing and feature extraction are used for cell recognition and automatic determination of characteristics (features) that describe the D antigen occurrence in cell populations. Based on the feature values non-linear machine learning algorithms are applied and result in the final Rhesus D type classification. Thus, this straightforward method holds great promise for reliable D antigen classification.

## Results

**Figure 1:**
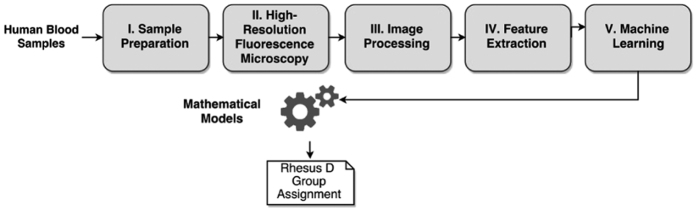
Schematic representation of the analysis workflow for the classification of Rhesus D types. (I). Blood samples are labelled with fluorescently marked antibodies and (II.) imaged by high-resolution fluorescence microscopy. (III.) Automatic cell detection and single molecule analysis is performed on acquired images. (IV.) Features are extracted and used for machine learning (V.) and the subsequent calculations of mathematical models, yielding a final Rhesus D type assignment.

We have established an effective combinatory workflow (depicted in Fig. 1) consisting of high-resolution fluorescence microscopy, image processing, and machine learning techniques. For Rhesus D type classification, 51 human blood test samples of already known Rhesus D types (namely D+, weak D, DEL, and D−; listed in Table 1) were measured and more than 2000 microscopy images (image size ~82*82 μm^2^) were analysed at single molecule and single cell level.

### High-resolution fluorescence microscopy of Rhesus D phenotypes

Three replicas of each sample were independently marked with Atto655-labelled antibodies H41 (Atto655-H41-Ab), BRAD3 (Atto655-BRAD3-Ab), and BIRMA-D6 (Atto655-BIRMA-D6-Ab) binding D antigen epitopes not affected in the analysed weak D and DEL types. Their use in immunohaematological routine anticipates that they have good binding affinities and accessibility to the corresponding epitopes. Fluorophore Atto655 was covalently attached to the antibodies (average degree of labelling of 1.8) and marked antibodies were applied in amounts to ensure saturation of all RhD molecules. In order to avoid false positive signals from antibodies sticking to the bottom and RhD immobilization caused by protein adhesion to the glass, the apical side of the erythrocytes was imaged. One-colour imaging using red-absorbing fluorophore Atto655 was applied in order to reduce autofluorescence and cytotoxic photodamage. Positive (D+ blood samples) and negative controls (D− blood samples) were carried along during each experiment. All amino acids changed in the weak D and DEL types analysed within this study are located in the intracellular or transmembrane part of the protein (Supplementary Fig. 1) and do not interfere with antibody-antigen interaction.

**Figure 2:**
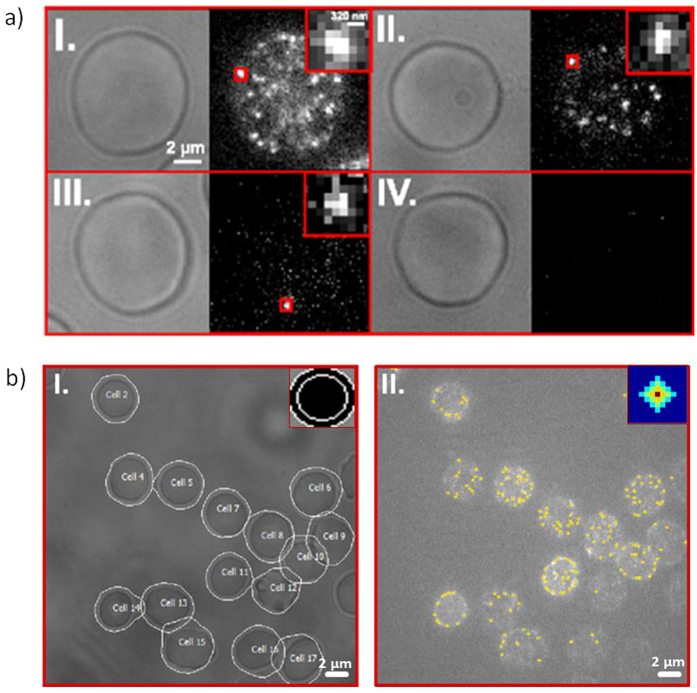
Exemplary microscopy images from four Rhesus D type populations differing in the amount of RhD protein incorporated into the upper erythrocyte cell membrane (a) and examples of erythrocyte and fluorescence peak detection (b). (**a**) (a.I.) D+ sample (RHD*01), (a.II.) weak D sample (RHD*weak D type 3), (a.III.) DEL sample (RHD*DEL8), (a.IV.) D− sample (RHD*01N.01). Atto655-H41 labelled D antigens correspond to the bright dots on the cells. Insets show individual fluorescence peaks. Images were recorded with 10 ms illumination time. (**b**) (b.I.) shows detected cell contours on a bright-field image and the used ring kernel. (b.II.) depicts all detected fluorescence peaks on the image and the used structuring element for detecting sphere structures in the image.

Cell contours of erythrocytes and intensity peaks corresponding to the RhD antigen were detected and analysed using the implemented automated image processing techniques. Parametrisation was done in a user-assisted fashion but was not changed during the experiments (see Methods for further details). Figure 2b.I. depicts the automatically detected contours of all erythrocytes from a bright-field image. Atto655-H41-Ab labelled D antigens correspond to the intensity peaks on the cells with intensities proportional to the count rates of fluorescence emission. The yellow dots in Fig. 2b.II mark the centres of the automatically detected peaks. All imaged cells are detected, regardless of their size and shape. Incompletely imaged cells, however, as for instance chopped cells on the edge of the images, are not considered in the subsequent analysis steps, as incomplete information about cells would bias all further statistical results and implications.

### Descriptive statistical analysis of fluorescence peak intensities

High-resolution fluorescence microscopy of blood samples revealed differences in the peak intensities between the four different RhD populations, namely D+, weak D, DEL, and D− (Fig. 2a). Figure 2a.I. shows a typical image of a cell obtained from a D+ sample, representative for all analysed samples (number of samples n = 12). Here, we use the peak intensities as a parameter that describes the cell populations and hints at the RhD clustering behaviour of different samples. Each peak intensity value has been calculated as the sum of intensities in a 3 × 3 pixel area around the peak maximum (Table 2). Analysis revealed average peak intensities (μ ± σ) of the D+ cell populations in the range of 11.7 ± 4.0*10^3^ counts/peak using Atto655-H41-Ab labelling, 7.6 ± 2.4*10^3^ counts/peak using Atto655-BRAD3-Ab labelling, and 7.0 ± 1.4*10^3^ counts/peak using Atto655-BIRMA-D6-Ab labelling. All D+ cells showed a fluorescence signal.

In total, we analysed 5894 cells using Atto655-H41-Ab (average cell count per sample 115), 5826 cells using Atto655-BRAD3-Ab (average cell count per sample 110), and 5731 cells using Atto655-BIRMA-D6-AB (average cell count per sample 111). Figure 2a.II. depicts a cell representative for a weak D sample (n = 14), in this case RHD*weak D type 3. All analysed cells in this population were labelled with at least one fluorescent antibody. Average peak intensities of all weak D cell populations are in the range of 7.4 ± 1.4*10^3^ counts/peak using Atto655-H41-Ab labelling, 6.3 ± 0.8*10^3^ counts/peak labelled with Atto655-BRAD3-Ab labelling, and 6.2 ± 0.6*10^3^ counts/peak using Atto655-BIRMA-D6-Ab labelling. Analysis of DEL cell populations (n = 12, Fig. 2a.III.), with only ~10% of all cells labelled, revealed average peak intensities in the range of 6.2 ± 1.0*10^3^ counts/peak using Atto655-H41-Ab labelling, 6.4 ± 0.7*10^3^ counts/peak labelled with Atto655-BRAD3-Ab labelling, and 6.2 ± 0.7*10^3^ counts/peak using Atto655-BIRMA-D6-Ab labelling. Figure 2a.IV. shows a cell representative for the D− cell population; for ~1% of the cells, sparsely distributed peaks were detected. The average peak intensities of all D− cell populations are in the range of 6.7 ± 2.0*10^3^ counts/peak using Atto655-H41-Ab labelling, 6.0 ± 0.7*10^3^ counts/peak using Atto655-BRAD3 labelling, and 6.0 ± 0.7*10^3^ counts/peak using Atto655-BIRMA-D6-Ab labelling.

In a separate experimental setting, we performed a statistical comparison of the distributions of individual Atto655 and single Atto655-marked antibodies on protein G coated glass to the very sparse signal occurrences on D− and DEL cells (Supp. Note including Supp. Figs 8 and 9,^37^,^38^). Statistical analyses revealed that the distribution of individual, Atto655 marked antibodies on coated glass and that of sparsely distributed antibodies on D− as well as on DEL cells have a high similarity. High average peak intensities of the fluorescently labelled D+ population indicate that a part of the signals originate from several fluorescent antibodies.

**Figure 3:**
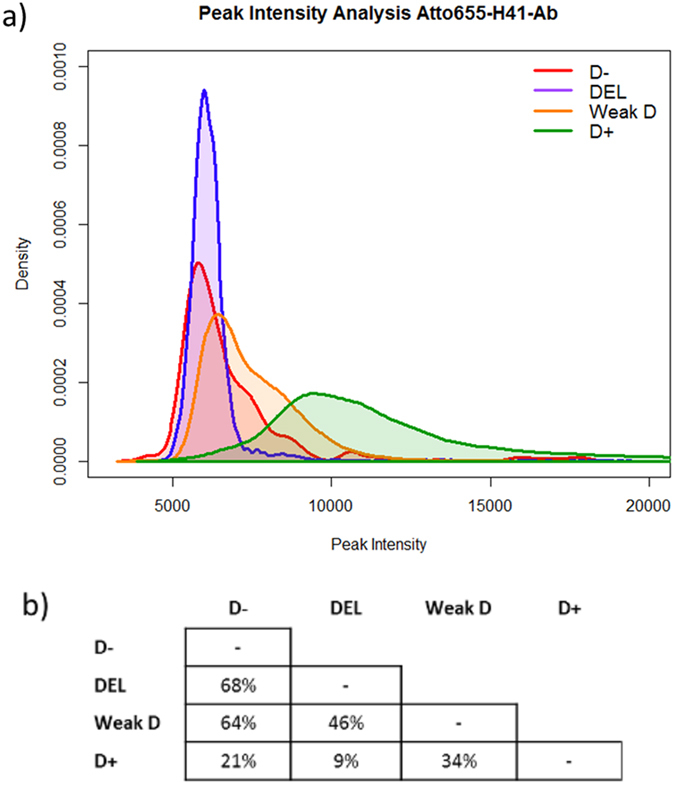
Distributions of peak intensity of each Rhesus D type using Atto655-H41-Ab labelling (a) and analysis of distribution overlaps (b). (**a**) A large overlap between all distributions of analysed RhD types is noticeable, especially for the D− (red) and DEL (blue) samples, but also for weak D (orange) and D+ (green) samples. (**b**) Results of the analysis of the distribution overlaps on acquired Atto655-H41-Ab dataset with respect to peak intensities; overlap percentages are calculated as the overlapping histogram area using a bin-size of 50. Large overlaps are observed between the Rhesus D types DEL and D−, weak D and D−, and weak D and DEL.

A simple comparison of the peak intensity distributions of different populations may lead to the incorrect conclusion that a Rhesus D type classification can be achieved using just a single parameter. The analysis of the intensity distributions shows large overlaps between individual populations, as for instance a 68% overlap between D− and DEL samples for Atto655-H41-Ab labelling or 70% for Atto655-BRAD3-Ab and Atto655-BIRMA-D6-Ab labelling. All calculated percentages of overlaps between the four populations are summarised in Fig. 3b and Supplementary Figs 2 and 3.

### Population differentiation by machine learning

Machine learning supported algorithms, however, are capable of automatic classification of such overlapping populations. Hence, Rhesus D blood group assignment can only be fulfilled by using more comprehensive information about the D antigen abundance on individual cells and cell populations. Therefore, several features based on single molecule information were used for machine learning. The following features were extracted: number of peaks, cell intensity, standard deviation of cell intensity, peak density, distance complete and nearest, and intensity ratio (Table 3). The feature cell intensity is of special interest, as this parameter is comparable to the parameters used in flow cytometry.

**Figure 4:**
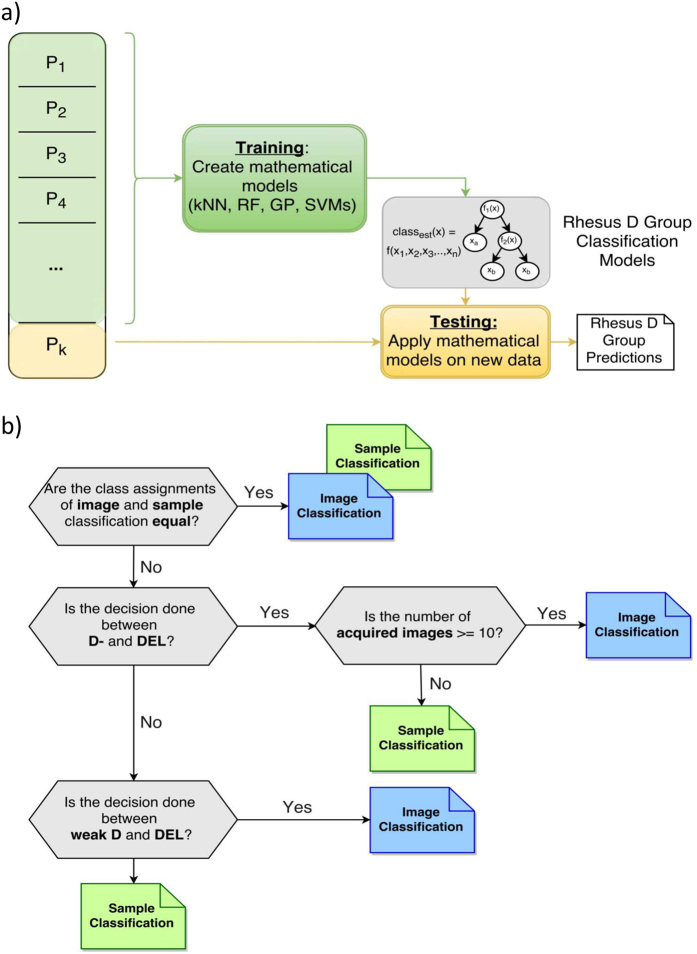
Workflow of a generic machine learning approach using k-fold cross validation (a) and workflow of the proposed combinatory classification method (b). (**a**) The used dataset is divided into *k* subsets; in each run one subset is reserved for testing purposes, all other subsets are used for training. After this process is repeated k-times, all results are gathered as the final classification result. (**b**) All classification results are based on image or on sample level in order to yield a final Rhesus D type assignment. The final assignment is made by processing all classification rules.

Subsequently, we applied machine learning on the extracted features in order to determine the Rhesus D type assignment of the sample. A schematic representation of the machine learning workflow is shown in Fig. 4a. A cross validation approach was used to ensure the reliability and accuracy of our results. The used dataset was split multiple times into training and testing partitions. The training subset was used to create mathematical models that can be considered as functions used to generate a classification vote out of given input parameters. The testing subset was used to test the previously created mathematical models on new data, as well as to assess the classification performance.

The classification task of the here analysed Rhesus D types is rather challenging, because large peak intensity overlaps between each population and a high heterogeneity within each population are present. In order to obtain a final Rhesus D type assignment, a combined classification method (see Methods) was implemented. This combinatory classification method comprises two different approaches, namely image level classification (method 1) and sample level classification (method 2).

Image level classification performed well for classification of weak D and DEL samples with accuracies of up to 92% for DEL and 83% for weak D using Atto655-BRAD3-Ab labelling, 75% for DEL and 92% for weak D using Atto655-BIRMA-D6-Ab labelling, and 100% for DEL and 92% for weak D using Atto655-H41-Ab labelling. However, the classification results for D+ and D− samples are not sufficient, as for instance none of the D− and only 33% of all D+ samples using Atto655-BRAD3-Ab labelling were correctly identified (Supplementary Tables 1, 2 and 3).

Sample level classification provides a higher overall classification accuracy compared to image level classification: 58% of all D+ and 53% of all D− samples were classified correctly using Atto655-Brad3-Ab labelling, 75% and 84% using Atto655-BIRMA-D6-Ab labelling, and 83% and 76% using Atto655-H41-Ab labelling. In contrast, sample level classification results for low expressed RhD types are worse compared to image level classification results.

Method 3 combines the advantages of method 1 (high classification accuracies for low expressed and highly heterogeneous cell population) and method 2 (high classification accuracies for common and rather homogenous cell population). Furthermore, classification rules are defined to determine the final Rhesus D type assignment by choosing between image and sample level classification results (see Methods, Fig. 4b).

This new method achieves higher classification accuracies, as for instance an overall test classification accuracy of 64% using Atto655-BRAD3-Ab labelling has been obtained. Hence, ten out of twelve (83%) DEL samples are classified correctly. Using Atto655-BIRMA-D6-Ab labelling, the majority of DEL samples are classified properly (83%) with an overall test classification accuracy of 78%. Best results are obtained using Atto655-H41-Ab labelling: A test classification accuracy of 96% has been observed. All D+ samples and all weak D samples are classified correctly. Furthermore, very low expressed DEL samples are all but one correctly classified; only one out of 13 D− samples is classified as DEL. A comprehensive result listing can be found in Table 4 and Supplementary Tables 1, 2, and 3.

## Discussion

We provide a comprehensive workflow for improved Rhesus D type classification. This classification is achieved by acquiring high-resolution fluorescence microscopy images, detecting single cells and fluorescence signals, extracting and calculating features from the given information, creating mathematical models, and finally applying the latter ones in order to get a final Rhesus D type assignment. We have developed multiple classification rules that enable a more accurate and sensitive Rhesus D type classification compared to commonly used laboratory methods by taking into account information on protein expression at single cell level.

Most accurate results for automatic Rhesus D type assignment were obtained using method 3 and Atto655-H41-Ab labelling. Here, 49 out of 51 human blood samples were classified correctly. Only one D− and one DEL sample were classified incorrectly, which yields an overall classification accuracy of 96%. In both cases only one method of the combinatory classification approach failed in correct assignment.

Comparison of the peak intensity distributions of Atto655-BIRMA-D6-Ab and Atto655-BRAD3-Ab labelled D+ and weak D samples reveals a larger overlap compared to Atto655-H41-Ab labelled samples (Supplementary Figs 2 and 3 compared to Fig. 3a). Consequently, the accuracy of Rhesus D type classification for D+ and weak D with Atto655-BIRMA-D6-Ab and Atto655-BRAD3-Ab labelling is reduced. In the case of Atto655-BRAD3-Ab and Atto655-BIRMA-D6-Ab labelled samples, the peak intensity distributions of all four RhD populations overlap, aggravating correct classification. The large overlaps of the peak intensity distributions lead to the assumption that the misassignment is of biochemical origin caused by differences in the accessibility of extracellular epitopes of the D antigen as well as diversity in antibody affinity to related protein motives^36^.

The analysis of the DEL type RHD*DEL8 (RHD IVS3+1 G>A) shows a surprising result: Körmöczi *et al.*^13^ briefly mentioned that binding of antibodies BRAD3 and BIRMA-D6 in this DEL variant has not been detected by adsorption-elution technique. This observation is not fully consistent with our results, since we observed signals for a part of the DEL population using the same antibody. We assume that this discrepancy can be explained by the higher sensitivity of our method. However, a more detailed analysis is beyond the scope of this contribution.

Our workflow can be used as a complementary method to standard immunohaematological techniques to reveal otherwise undetected very low expressed Rhesus D types, which have a prevalence of up to 30% in Asian D− population^11^. Whereas commonly used adsorption-elution technique lacks standardised protocols, is time-consuming and requires an experienced technician, our method is less laborious. The substantially shorter incubation time (30 minutes compared to several hours) of blood cells with a high concentration of antibodies and the shorter washing procedure both save time. Moreover, less experience is required for sample preparation as well as fluorescence microscopy. The developed machine learning based analysis software performs feature calculation and RhD phenotype classification automatically. Of additional advantage are the visualisation of individual antigens on cells without limitations on sample size and the visual control of detected antigens used for analysis. Actually, the quality of separation of the cell populations of different Rhesus D types is in our case only limited by the biochemistry of the applied antibodies.

Here, we have shown that our method achieves a reliable discrimination of well described RhD subpopulations. The high sensitivity of our method revealed intra-population variability, which has yet not been observed and hence represents a new form of blood group typing. The application of standard antibodies facilitates the straightforward implementation of our technique in immunohaematological routine. Since high throughput methods for expression level analyses (e.g. *RHD* typing) are gaining in importance^18^,^22^, we also suggest the use of multi-colour labelling and implementation of a high speed imaging system (e.g. a nanoreader^39^,^40^) to accelerate RhD type classification.

The presented method can be used to characterise the expression level of novel *RHD* alleles or to validate new methods in which determination of very low levels of protein expression is essential^41^. This technique holds great promise to improve the safety of red blood cell units and to prevent dangerous transfusion incidents. Moreover, this workflow is broadly applicable in a variety of scientific fields, such as in molecular biology and medicine (in cases of cell population classification by rarely expressed cell markers) as well as in biophysics and material science.

## Methods

### Blood samples

Ethylendiaminetetraacetate (EDTA)-anticoagulated blood samples were provided by the Red Cross Transfusion Service (Linz, Upper Austria, Austria). RhD assignment was done by standard serology^24^ and *RHD* gene sequencing was performed on samples with weak D and DEL phenotypical expression. The sample cohort (Table 1) consisted of the most common weak D types in Europe, RHD*weak D type 1 (n = 6), RHD*weak D type 2 (n = 3), and RHD*weak D type 3 (n = 5), two *RHD* alleles causing DEL phenotypical expression, RHD*DEL8 (n = 6), and RHD*09.05 (n = 6). D+ (n = 12) and D− control samples (n = 13) were provided for each analysis. Red blood cells were prepared within 7 days of sampling.

### Statement on the use of human blood samples

All human blood samples were kindly provided by the Red Cross Transfusion Service (Linz, Upper Austria, Austria) and were collected during routine blood donations in accordance with the strict policies of the Red Cross Transfusion Service Linz. The usage of residual blood material from blood donations is, as captured in a written consent of the Upper Austrian Ethic Commission, not subject of the Austrian Tissue Safety Act. Nevertheless, all blood donors signed their informed consents that potential residual blood material can be used for research and development purposes. All experimental protocols were approved by and carried out in collaboration with the Red Cross Transfusion Service Linz.

### Immunohaematology

All samples were incubated using monoclonal antibodies targeting different epitopes of the D antigen: Atto655-H41-Ab (binds to epitope 3.1), Atto655-BRAD3-Ab (binds to epitope 6.2), and Atto655-BIRMA-D6-Ab (binds to epitope 9.1). Atto655-H41-Ab was generously supplied by Bio-Rad (Dreieich, Germany). Atto655-BRAD3-Ab and Atto655-BIRMA-D6-Ab were obtained from the International Blood Group Reference Laboratory (Bristol, UK).

### Antibody labelling

The primary antibodies were labelled independently via an N-hydroxysuccinimid (NHS)-ester with Atto655 (ATTO-TEC, Siegen, Germany): Atto655 was dissolved in anhydrous dimethylsulfoxid to yield a final concentration of 1 mg/mL. Monoclonal antibodies were mixed with Atto655 in 0.2 M sodium bicarbonate buffer at pH 8.4. An average degree of labelling of 1.8 ensures a high amount of antibodies with a single fluorophore molecule attached. The reaction mixture was incubated for 1 hour at room temperature. In order to remove unbound dye, gel filtration was applied using PD-10 SephadexTM G-25M columns (GE Healthcare, Buckinghamshire, UK). Fluorescently labelled antibodies were concentrated by cut-off filters (several centrifugation steps at 1500 g for 3 minutes with Vivaspin 6, MWCO: 10,000, Sartorius Stedim Biotech, Goettingen, Germany), aliquoted and stored at −20 °C.

### Sample preparation

100 μL EDTA-anticoagulated blood samples were washed with sodium chloride (0.9%, Fresenius Kabi Austria, Linz, Austria) at 79 g for 3 minutes. Erythrocytes were incubated for 30 minutes at 37 °C with antibodies in ID-CellStab (buffer specially formulated for erythrocytes; Bio-Rad Laboratories, Cressier, Switzerland). Unbound antibodies were removed by washing three times with sodium chloride. Subsequently, cells were resuspended in ID-CellStab.

### Fluorescence microscopy and image acquisition

Images were acquired with a modified Olympus IX81 inverted epifluorescence microscope, using a two axis scanning stage and an Olympus UAPON 100x/1.49 NA oil objective. Blood samples were illuminated with a diode laser at 642 nm (Omicron-laserage Laserprodukte GmbH, Phoxx 642, Rodgau-Dudenhofen, Germany). The signal was acquired using an Andor iXonEM+ 897 (back-illuminated) EMCCD (16 μm pixel size). The following filter sets were used: Dichroic filter (ZT405/488/561/640rpc, Chroma, Olchin, Germany), emission filter (446/523/600/677 nm BrightLine quad-band band-pass filter, Semrock, Rochester), and an additional emission filter (HQ 700/75 M, NC209774, Chroma Technology GmbH, Olching, Germany). The signal was acquired for 10 ms with 50 ms delay at 0.75 kW/cm^2^ excitation power. Conversion of fluorescence intensities into photon counts is given by: 1 count/pixel = 0.3 photons/pixel.

**Figure 5:**
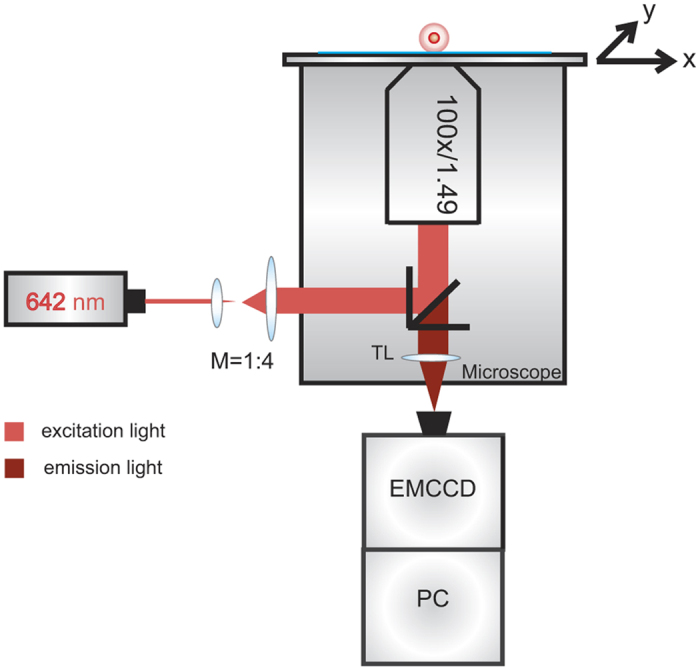
Sketch of the fluorescence microscope used for analysis of Rhesus D protein abundance on erythrocytes. The laser beam produced by a diode laser (642 nm) is expanded 4:1 via a telescope and focused into the focal plane by an oil-immersion, infinity corrected objective (100x magnification with a high numerical aperture). In the detection path, fluorescence light is focused via a tube lens (TL) on an EMCCD camera. Images were recorded with 10 ms illumination time.

The signal-to-noise ratio was 31 ± 9. An image sequence of 150 images was recorded. The first ten images were acquired using bright field microscopy to enable assignment of fluorescence signals to distinct cells. The illumination protocols were performed with custom-made LabView-based control software. All samples were measured within 24 hours since results of test experiments (data not shown) proved that fluorescence intensities remained constant within this time period: For D+ samples the peak intensity variation between measurements at days 1 and 2 was 2 ± 10%, for DEL samples 1 ± 19%, and for weak D samples 6 ± 17%; for D− samples no change was measured. A sketch of the fluorescence microscopy setup can be found in Fig. 5.

### Data analysis – cell and single molecule detection

All data analysis tasks were performed using implemented and adapted image processing techniques. For cell detection tasks we applied thresholding, mean filtering, convolution, evolution strategies, and an active contour method^42^,^43^,^44^,^45^,^46^. Those methods allow detection of all erythrocytes in each image, regardless of their shape or size. For molecule detection (D antigen occurrences on the cell membrane) conservative smoothing, top-hat filtering, thresholding, and region growing were applied^47^,^48^. Details on the used image processing methods and the used parameterisations can be found in the Supplementary Material. The so developed analysis framework, a short documentation, and exemplary microscopy images can be found on the Bioinformatics Research Group homepage (http://bioinformatics.fh-hagenberg.at/site/index.php?id=16).

### Feature definition and extraction

The identification of cell contours enables the assignment of fluorescence signals to the corresponding erythrocyte. Based on this assignment, further statistical analyses at the cell level (considering data of individual cells) as well as at the image level (considering all cells per image) were performed. Features that include information obtained at the cellular and molecular level were used to distinguish between different D antigen types. In Table 3 all extracted features and their short explanations are listed in detail. A detailed explanation and calculation formulas can be found in the Supplementary Information.

These features further serve as input for machine learning methods that are used to learn models which classify samples according to their Rhesus D type. Boxplots for each feature, showing their distributions and mean values among the analysed Rhesus D types, are depicted in Supplementary Fig. 7.

### Statistical analyses

Statistical analyses were carried out using R^49^ and the main implemented statistical functionalities. Data sorting and filtering was done using Microsoft Excel. Distribution plots were generated using the R ggplot package. If not stated otherwise all data is expressed as the mean ± SD.

### Peak intensity distribution overlap

For each RhD type we calculated the average peak intensity distributions of all analysed samples. The peak intensity has been calculated as the sum of intensities in a 3 × 3 pixel area around the peak maximum. Figure 3a and Supplementary Figs 2 and 3 show the peak intensity distributions for samples labelled with Atto655-H41-Ab, Atto655-BIRMA-D6-Ab, and Atto655-BRAD3-Ab. If the distributions were clearly separated from each other, then this feature would be sufficient for a clear Rhesus D type identification.

For calculating the overlap of distributions we split the intensity range into bins of size 50. For each pair of Rhesus D types we extracted the overlapping area of each bin by extracting the minimum of the two values. The sum of all detected minima of each bin reflects the overlapping percentages of these two Rhesus D types.

### Machine learning algorithms

In general, data mining is understood as the practice of automatically searching for patterns in large stores of data. In order to do so, a set of input parameters and a set of target variables are defined and further used to create a mathematical model. The generation of a mathematical model is done by machine learning algorithms. In the here presented study classification algorithms were used to generate mathematical models that are able to classify samples on the basis of their features^50^.

Here, all tasks were performed as classification tasks using the implementation in the HeuristicLab framework^51^. The following classification algorithms were applied: random forests (RFs^52^,^53^), support vector machines (SVMs^54^), genetic programming with offspring selection (GP^50^,^55^,^56^), and k-nearest neighbour classification (kNN^57^). Further details can be found in the Supplementary Materials.

Each algorithm was performed using 5-fold cross validation and was repeated multiple times (n = 40), which results in multiple classification models that are combined via majority voting. The majority voting was performed by counting the votes of all mathematical models for each Rhesus D type separately. Afterwards, the final assignment was made by selecting the class with the majority of votes^58^. Thus, images or cells without signals had a lower impact on the overall classification result.

### Method 1: Image level classification

Classification method 1 implemented image level based classification by classifying all images separately according to their Rhesus D type assignment. All images and the corresponding extracted features were used as input. For each image 160 (each algorithm is repeated 40 times for each image) mathematical models (classifiers) were created, and each model votes for a certain Rhesus D type. Subsequently, we used a majority voting step which collects all classification statements (votes) of all images from each sample. A final classification for each sample was made via the majority of votes. This method renders a robust class assignment for low expressed Rhesus D phenotypes, since more information on cell population heterogeneity is captured.

### Method 2: Sample level classification

Sample level classification was based on the averages of the feature values of all images from one sample. This new dataset was used to create 160 mathematical models that vote for certain Rhesus D types. The final assignment for each sample was made by choosing the Rhesus D type with the majority of votes. The sample level classification allowed distinguishing RhD types with homogenous cell populations.

### Method 3: Combinatory classification based on sample and image level information

Method 3 is based on sample and image level information and combines the advantages of method 1 and 2. Method 1 performs best for heterogeneous cell populations; method 2 enables a robust classification for homogenous cell populations. For this purpose, method 1 and method 2 were applied (independently from each other) and all classification results were stored. Subsequently, classification rules were defined to regulate the class assignment process between the classification results of methods 1 and 2:

- First the concordance of both classification methods had to be examined. If both classification results were concordant, the sample was assigned to this class.
- Otherwise the involved classes were further analysed:

- If the decision had to be made between D− and DEL samples, the two most similar classes, the decision was based on the number of images acquired for the specific sample.
  - If there were at least 11 images, the image classification result was chosen as we considered this information enough for a reliable class assignment.
  - If there were fewer images, it was more reliable to choose the classification result at the sample level.
- Additional rule for weak D and DEL classification:
  - For differentiation between weak D and DEL, the classification result of method 1 was chosen, as here more information about cell heterogeneity and image heterogeneity is included.

In any other case, the result of method 2 was the assignment of choice.

## Additional Information

**How to cite this article**: Borgmann, D. M. *et al.* Single Molecule Fluorescence Microscopy and Machine Learning for Rhesus D Antigen Classification. *Sci. Rep.* **6**, 32317; doi: 10.1038/srep32317 (2016).

## Supplementary Material

Supplementary Information

## Figures and Tables

**Table 1:** Overview of analysed *RHD* types.

D phenotype	Allele Name	Expression Level	Molecular Information	Number of Analysed Samples
D+	RHD*01	High expression	Reference sequence	12
weak D	RHD*weak D type 1	Low expression	Single missense mutationc.809T>G (V270G)	6
	RHD*weak D type 2		Missense mutation (splice site affectedc.1154G>C (G385A))	3
	RHD*weak D type 3		Single missense mutationc.8C>G (S3C)	5
DEL	RHD*09.02 Weak D type 4.3	Very low expression	multiple missense mutationsc.602C>G (T201R),c.667T>G (F223V),c.819G>A,c.872C>G (P291R)	6
	RHD*DEL8 (IVS3+1G>A)		Splice site mutation	6
D−	RHD*01N.01	No expression	Complete *RHD* gene deletion	13
Partial D	*/*	Type dependent	Mutations in the extracellular part, geneconversion (hybrid alleles)	0

Their allele name, molecular information, and the number of analysed samples (n = 51).

**Table 2:** Statistics of the average peak intensities.

Atto655-BRAD3-Ab	μ	σ
D+	7.6*10^3^	2.4*10^3^
D−	6.0*10^3^	0.7*10^3^
DEL	6.4*10^3^	0.7*10^3^
Weak D	6.3*10^3^	0.8*10^3^
**Atto655-BIRMA-D6-Ab**		
D+	7.0*10^3^	1.4*10^3^
D−	6.5*10^3^	0.6*10^3^
DEL	6.2*10^3^	0.7*10^3^
Weak D	6.2*10^3^	0.6*10^3^
**Atto655-H41-Ab**		
D+	11.7*10^3^	4.0*10^3^
D−	6.7*10^3^	2.0*10^3^
DEL	6.2*10^3^	1.0*10^3^
Weak D	7.4*10^3^	1.4*10^3^

(μ and σ, assigned as counts per peak) of all Rhesus D types using Atto655-BRAD3-Ab, Atto655-BIRMA-D6-Ab, and Atto655-H41-Ab labelling.

**Table 3:** Detailed list of features used for machine learning in order to assign Rhesus D types to human blood samples.

Feature	Explanation
number of peaks	average number of detected peaks on each image
cell intensity	average intensity of all detected peaks per cell
standard deviation of cell intensity	variability between average intensities of all detected peaks per cell
peak density	average density of detected peaks per cell
distance complete	average distance between all peaks within a cell
distance nearest	average distance between nearest peaks within a cell
intensity ratio	average intensity ratio between intra-cell and inter-cell areas

**Table 4:** Classification results based on method 3.

		Actual				
Atto655-BRAD3-Ab		D+	D−	DEL	Weak D	
Predicted	D+	50%	0	0	0	
	D−	0	**38.46%**	8.34%	0	
	DEL	8.33%	61.54%	**83.33%**	14.29%	
	Weak D	41.67%	0	8.33%	**85.71%**	
						**64%**
		**Actual**				
**Atto655-BIRMA-D6-Ab**		**D+**	**D−**	**DEL**	**Weak D**	
Predicted	D+	50%	0	0	0	
	D−	0	**84.62%**	0	0	
	DEL	0	15.38%	**83.33%**	7.14%	
	Weak D	50%	0	16.67%	**92.86%**	
						**78%**
		**Actual**				
**Atto655-H41-Ab**		**D+**	**D−**	**DEL**	**Weak D**	
Predicted	D+	**100%**	0	0	0	
	D−	0	**92.31%**	0	0	
	DEL	0	7.69%	**91.67%**	0	
	Weak D	0	0	8.33%	**100%**	
						**96%**

Overall test classification accuracies of 64% for Atto655-BRAD3-Ab, 78% for Atto655-BIRMA-D6-Ab, and 96% for Atto655-H41-Ab labelling are achieved.
