# Supplementary material for: Single Molecule Fluorescence Microscopy and Machine Learning for Rhesus D Antigen Classification
Source: Sci Rep. 2016 Sep 1;6:32317. doi: 10.1038/srep32317 (PMC5007495; doi:10.1038/srep32317)
Supplement: Supplementary Information [file srep32317-s1.pdf]

Supplementary Information:

Single Molecule Fluorescence Microscopy and Machine Learning for Rhesus D Antigen Classification

Daniela Borgmann*[1], Sandra Mayr*[2], Helene Polin[3], Susanne Schaller[1], Viktoria Dorfer[1], Lisa Obritzberger[1], Tanja Endmayr[2], Christian Gabriel[3], Stephan Winkler[1], Jaroslaw Jacak[2*]

The Supplementary Information includes:

Supplementary Figures 1-7

Supplementary Tables 1-3

Supplementary Methods

Supplementary Note including Supp. Figures 8 and 9

Supplementary Figures & Tables:

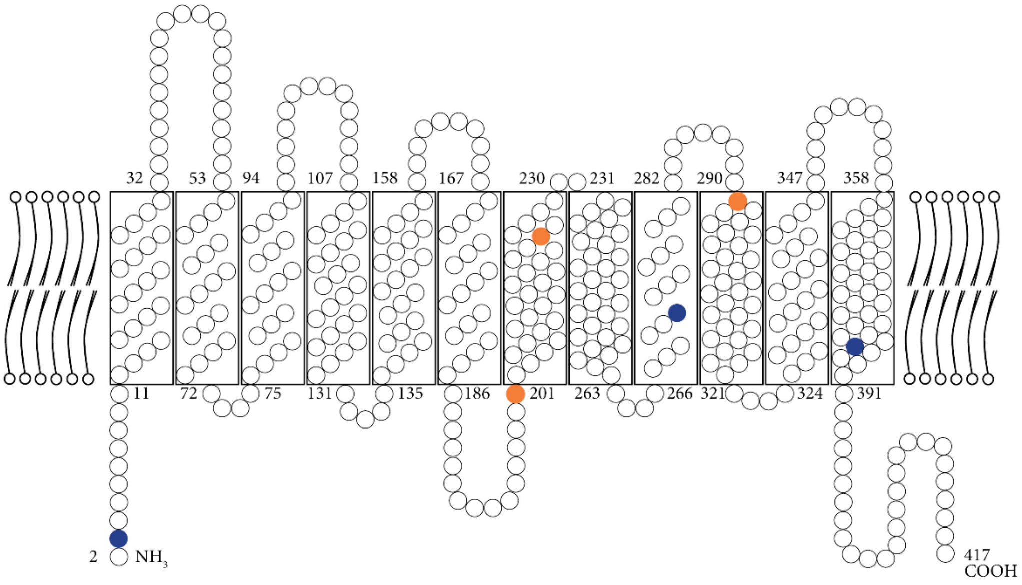

Supplementary Figure 1: Model of the RhD protein incorporated into the plasma membrane of erythrocytes. Four hundred and seventeen amino acids are arranged into twelve transmembrane helices and six extracellular loops harbouring more than thirty immunogenic epitopes. Amino acids affected in *RHD*weak D type 1*, *type 2* and *type 3* (blue circles) and those involved in DEL phenotype *RHD*09.05* (*RHD*weak D type 4.3*, orange circles) are located in the intracellular or transmembrane part of the protein (adapted from Wagner 2002[16]).

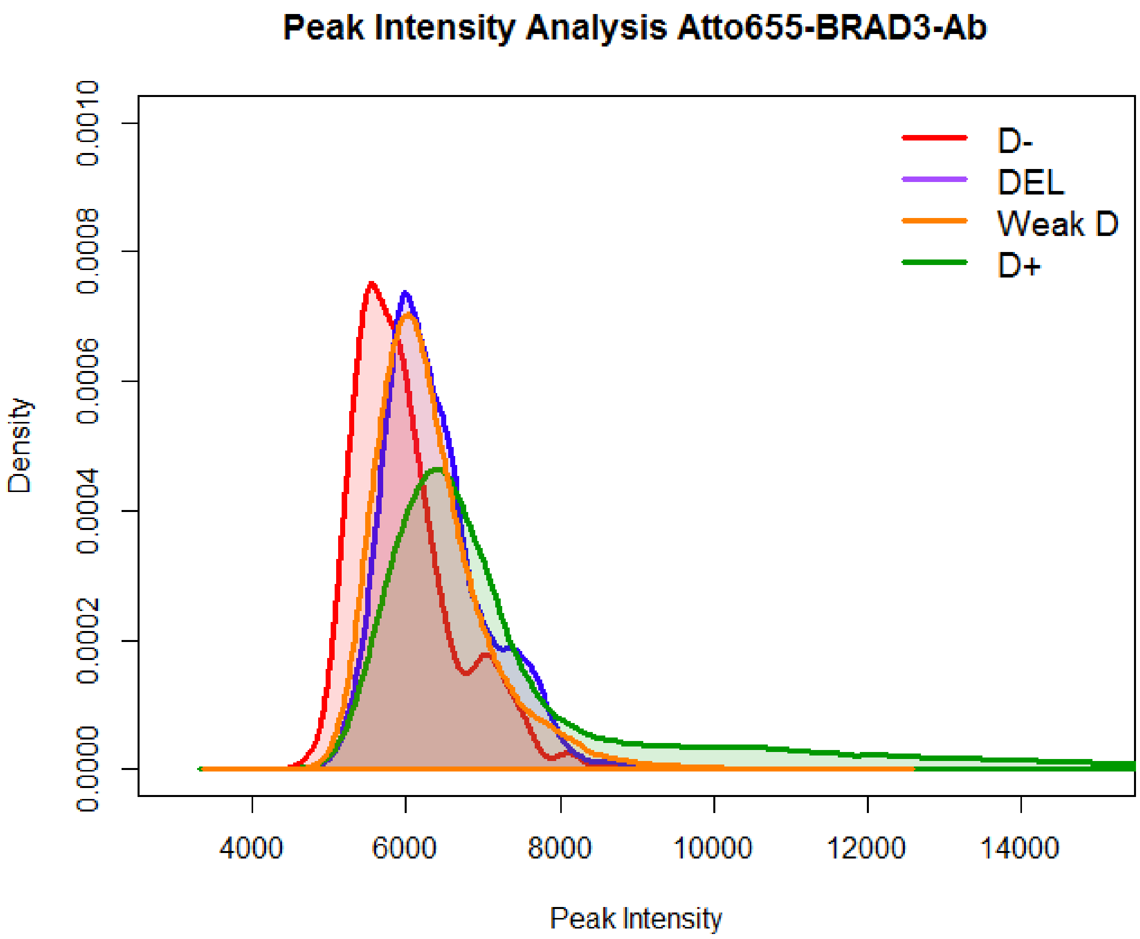

	D-	DEL	Weak D	D+
**D-**	-			
**DEL**	70%	-		
**Weak D**	76%	92%	-	
**D+**	55%	75%	72%	-

Supplementary Figure 2: (I). Distributions of peak intensity of each Rhesus D type using Atto655-BRAD3-Ab labelling. Large overlapping areas can be observed between all Rhesus D blood group types. **(II.) Analysis of distribution overlaps.** Results of the analysis of the distribution overlaps on acquired Atto655-BRAD3-Ab dataset with respect to peak intensities; overlap percentages are calculated as the overlapping histogram area using a bin-size of 50. Large overlaps are observed between the Rhesus types DEL and D-, weak D and D-, and weak D, and DEL.

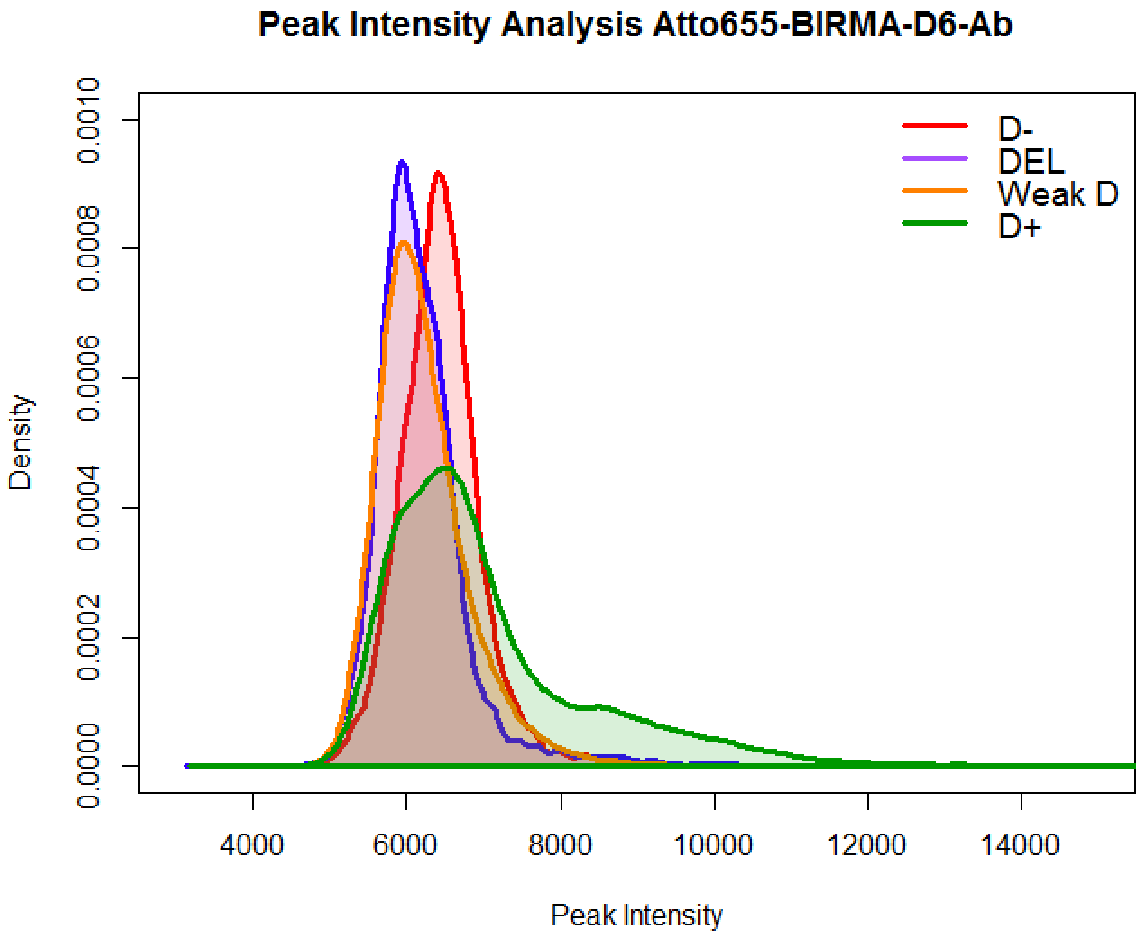

	D-	DEL	Weak D	D+
**D-**	-			
**DEL**	70%	-		
**Weak D**	73%	92%	-	
**D+**	71%	62%	68%	-

Supplementary Figure 3: (I). Distributions of peak intensity of each Rhesus D type using Atto655-BIRMA-D6-Ab labelling. Large overlapping areas can be observed between all Rhesus D blood group types. **(II.) Analysis of distribution overlaps.** Results of the analysis of the distribution overlaps on acquired Atto655-BIRMA-D6-Ab dataset with respect to peak intensities; overlap percentages are calculated as the overlapping histogram area using a bin-size of 50. Large overlaps are observed between the Rhesus types DEL and D-, weak D and D-, and weak D, and DEL.

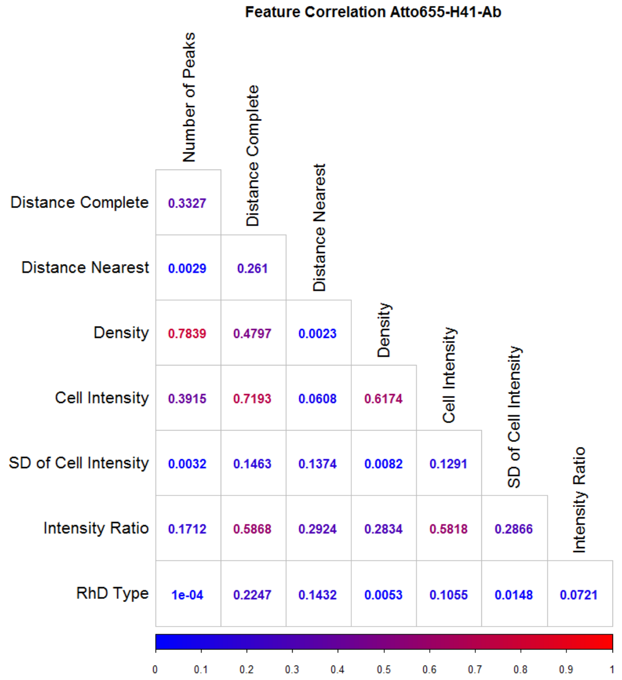

Supplementary Figure 4: Correlation matrix of the extracted features used for machine learning using Atto655-H41-Ab. Correlation values are given as Pearson's R^2 correlation values and were calculated using the Atto655-H41-Ab dataset. Red coloured values indicate high correlations; blue values indicate a low correlation.

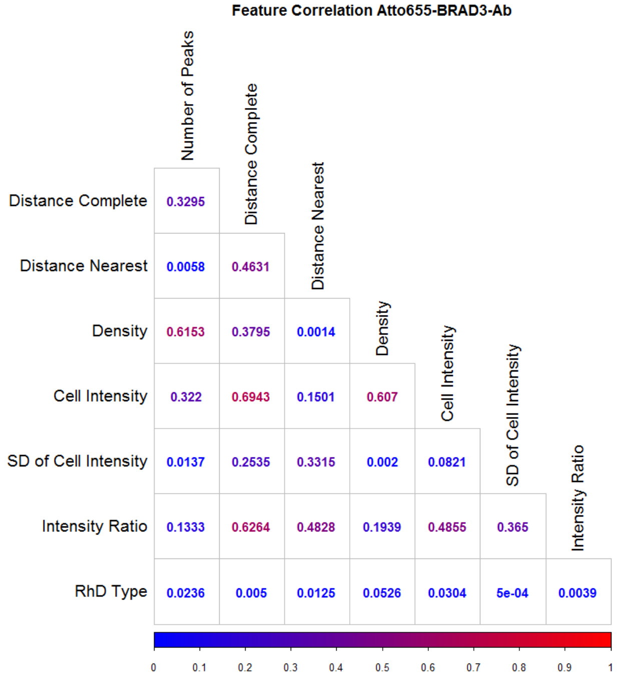

Supplementary Figure 5: Correlation matrix of the extracted features used for machine learning using Atto655-BRAD3-Ab. Correlation values are given as Pearson's R^2 correlation values and were calculated using the Atto655-BRAD3-Ab dataset. Red coloured values indicate high correlations; blue values indicate a low correlation.

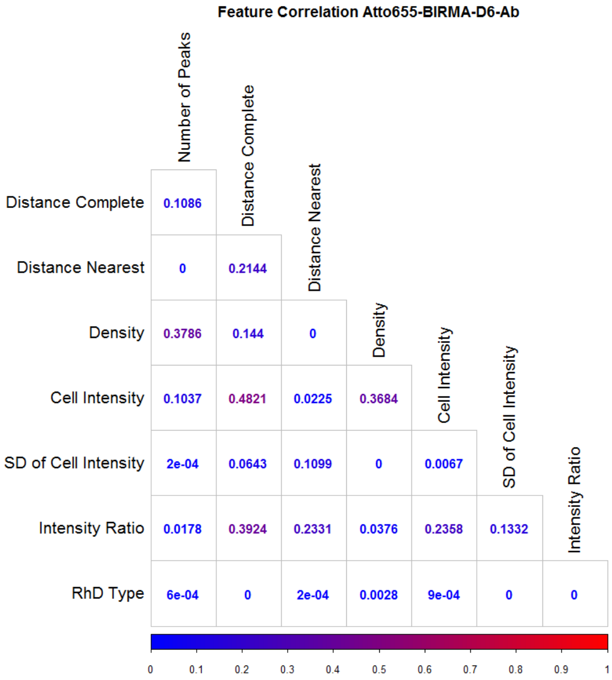

Supplementary Figure 6: Correlation matrix of the extracted features used for machine learning using Atto655-BIRMA-D6-Ab. Correlation values are given as Pearson's R^2 correlation values and were calculated using the Atto655-BIRMA-D6-Ab dataset. Red coloured values indicate high correlations; blue values indicate a low correlation.

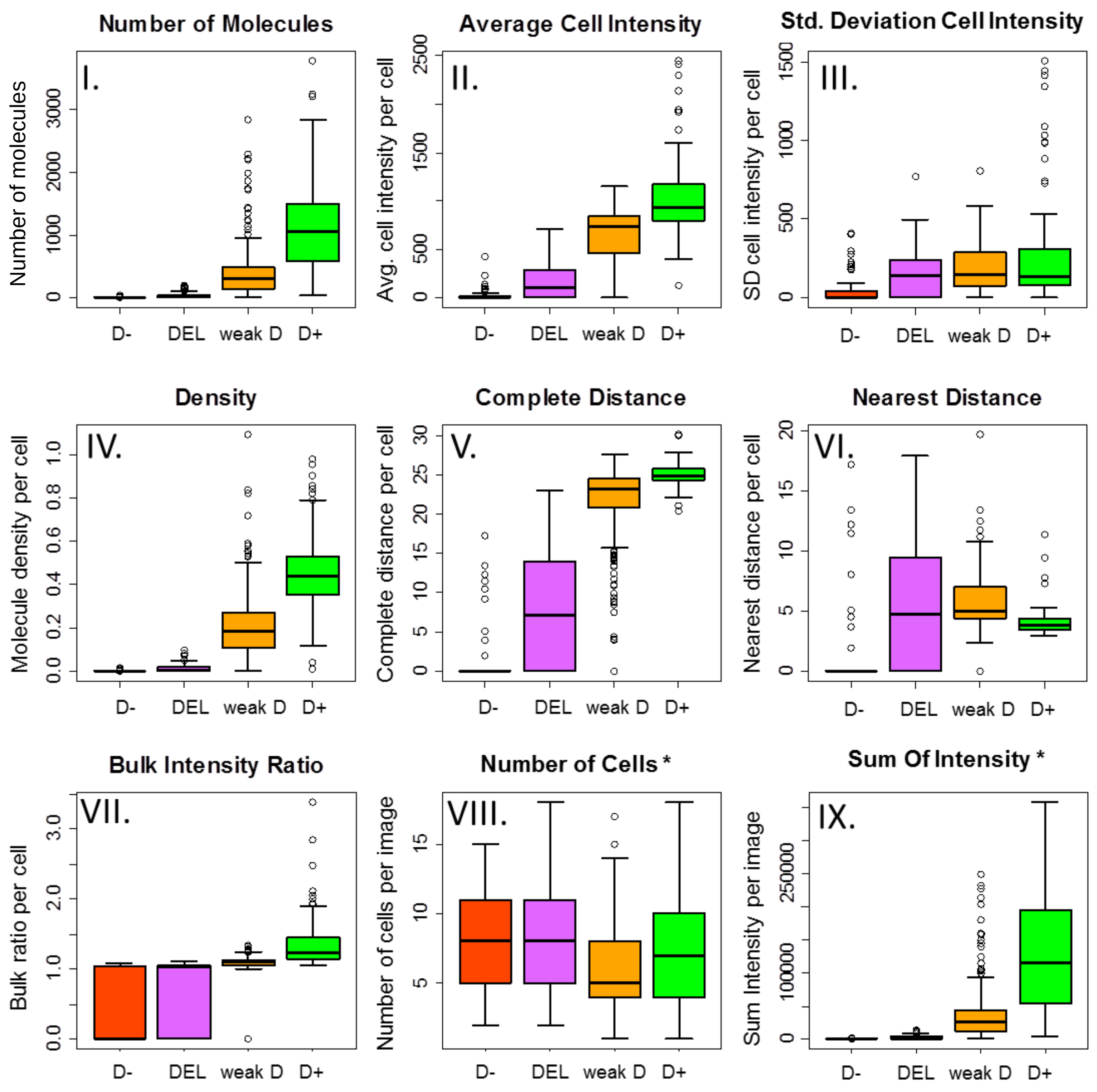

Supplementary Figure 7: Collection of extracted and calculated features and their distributions among the different Rhesus D types. Features depicted in **Suppl. Figure 4.I.-VII.** are used as input for machine learning approaches. **Suppl. Figure 4.VIII.** and **IX.** show features not used for machine learning but calculated for data completeness (average number of analysed erythrocytes on each image per Rhesus D type (**VIII**.) and sum of peak intensities per image per Rhesus type (**IX.**)). The four Rhesus D types, namely D-, DEL, weak D, and D+ are color-coded in this order: red, purple, orange, green.

Method 1

		Actual			
		D+	D-	DEL	Weak D
Predicted	D+	**33.33%**	0	0	0
	D-	0	**0**	0	0
	DEL	8.33%	100%	**83.33%**	7.14%
	Weak D	58.34%	0	16.67%	**92.86%**

52%

Method 2

		Actual			
		D+	D-	DEL	Weak D
Predicted	D+	**58.33%**	0	0	7.14%
	D-	0	**53.85%**	41.67%	0
	DEL	8.34%	46.15%	**50%**	14.29%
	Weak D	33.33%	0	8.33%	**78.57%**

60%

Method 3

		Actual			
		D+	D-	DEL	Weak D
Predicted	D+	**50%**	0	0	0
	D-	0	**38.46%**	8.34%	0
	DEL	8.33%	61.54%	**83.33%**	14.29%
	Weak D	41.67%	0	8.33%	**85.71%**

64%

Supplementary Table 1: Summary of classification results for Atto655-BRAD3-Ab labelling: Altogether 51 samples and 812 images were classified using classification methods 1, 2, and 3 yielding classification accuracies of 52%, 60%, and 64%.

Method 1

Predicted		Actual			
		D+	D-	DEL	Weak D
Predicted	D+	**41.67%**	0	0	0
	D-	0	**30.77%**	0	0
	DEL	0	69.23%	**75%**	7.14%
	Weak D	58.33%	0	25%	**92.86%**
					**60%**

Method 2

Predicted		Actual			
		D+	D-	DEL	Weak D
Predicted	D+	**75%**	0	0	35.72%
	D-	0	**84.62%**	16.67%	0
	DEL	0	15.38%	**58.33%**	28.56%
	Weak D	25%	0	25%	**35.72%**
					**62%**

Method 3

Predicted		Actual			
		D+	D-	DEL	Weak D
Predicted	D+	**50%**	0	0	0
	D-	0	**84.62%**	0	0
	DEL	0	15.38%	**83.33%**	7.14%
	Weak D	50%	0	16.67%	**92.86%**
					**78%**

Supplementary Table 2: Summary of classification results for Atto655-BIRMA-D6-Ab labelling: Altogether 51 samples and 812 images were classified using classification methods 1, 2, and 3 yielding classification accuracies of 60%, 62%, and 78%.

Method 1

		Actual			
		D+	D-	DEL	Weak D
Predicted	D+	**83.33%**	0	0	0
	D-	0	**30.77%**	0	0
	DEL	0	69.23%	**100%**	7.14%
	Weak D	16.67%	0	0	**92.86%**

75%

Method 2

		Actual			
		D+	D-	DEL	Weak D
Predicted	D+	**83.33%**	0	0	7.14%
	D-	0	**76.92%**	33.33%	0
	DEL	0	23.08%	**66.67%**	7.15%
	Weak D	16.67%	0	0	**85.71%**

78%

Method 3

		Actual			
		D+	D-	DEL	Weak D
Predicted	D+	**100%**	0	0	0
	D-	0	**92.31%**	0	0
	DEL	0	7.69%	**91.67%**	0
	Weak D	0	0	8.33%	**100%**

96%

Supplementary Table 3: Summary of classification results for Atto655-H41-Ab labelling: Altogether 51 samples and 793 images were classified using classification methods 1, 2, and 3 yielding classification accuracies of 75%, 78%, and 96%.

Supplementary Methods:

Feature Definitions

Predefined equations:

$numPeaks(img)$, represents the number of detected peaks in image img

$imgs(imgSeq)$, represents the collection of images of a given image sequence $imgSeq$

$peaks(c, img)$, represents the collection of peaks in a given cell c in image img

$intensity(p)$, represents the maximum intensity value of a given peak p

$cells(img)$, represents the collection of cells in image img

$area(c) = area(c, img)$, represents the area of the given cell c in bright-field image img

$euclideanDistance(p, q)$, represents the Euclidean distance between two points calculated as $\sqrt{\sum_{i=1}^{n}(q_i - p_i)^2}$

$cellBulkIntensity(c, img)$, represents the sum of inter-cell pixel values per cell c on a given image img

$backgroundIntensity(img)$, represent the sum of intra-cell pixel values on a given image img

The following features were defined:

- number of peaks ($numPeaks$), calculated as the average number of detected peaks per image

$$\boldsymbol{numPeaks(imgSeq)} = \frac{\sum_{\boldsymbol{img} \in \boldsymbol{imgs(imgSeq)}} \boldsymbol{numPeaks(img)}}{|\boldsymbol{imgs(imgSeq)}|}$$

- cell intensity ($cellIntensity$), calculated as the average cell intensity of all cells per image

$$cellIntensity(c, img) = \frac{\sum_{peak \in peaks(c,img)} intensity(peak)}{|peaks(c, img)|}$$

$$cellIntensity(img) = \frac{\sum_{c \in cells(img)} cellIntensity(c, img)}{|cells(img)|}$$

$$cellIntensity(imgSeq) = \frac{\sum_{img \in imgs(imgSeq)} cellIntensity(img)}{|imgs(imgSeq)|}$$

- standard deviation of cell intensity ($stdCell$), calculated as the variability between cell intensities

$$cellIntensity(imgSeq)[i] = cellIntensity(imgSeq[i])$$

$$stdCell(imgSeq) = std(\{cellIntensity(img): img \in imgSeq\})$$

- peak density ($dens$), calculated as the average density of peaks per cell area

$$\boldsymbol{dens(c, img)} = \frac{|\boldsymbol{peaks(c,img)}|}{\boldsymbol{area(c)}}$$

$$\boldsymbol{dens(img)} = \frac{\sum_{\boldsymbol{c:c \in cells(img)}} \boldsymbol{dens(c, img)}}{|\boldsymbol{cells(img)}|}$$

$$dens(imgSeq) = \frac{\sum_{img:img \in imgs(imgSeq)} dens(img)}{|imgs(imgSeq)|}$$

- distance complete ($distanceComplete$), calculated as the average distance between all peaks within a cell

$$distanceComplete(peaks) = \frac{\sum_{(i,j):\, i \in \text{ peaks, } j \in \text{ peaks, } i \neq j} euclideanDistance(i, j)}{|\{(i, j) : i \in \text{ peaks, } j \in \text{ peaks, } i \neq j\}|}$$

$$\boldsymbol{distanceComplete(img)} = \frac{\sum_{\boldsymbol{c:c \in cells(img)}} \boldsymbol{distanceComplete(peaks(c, img))}}{|\boldsymbol{cells(img)}|}$$

$$distanceComplete(imgSeq) = \frac{\sum_{img:img \in imgs(imgSeq)} distanceComplete(img)}{|imgs(imgSeq)|}$$

- distance nearest ($distanceNearest$), calculated as the average distance between nearest peaks within a cell

$$distanceNearest(peaks) = \min_{(i,j):\, i \in \text{ peaks, } j \in \text{ peaks, } i \neq j}(\text{ euclideanDistance}(i, j))$$

$$\boldsymbol{distanceNearest(img)} = \frac{\sum_{\boldsymbol{c:c \in cells(img)}} \boldsymbol{distanceNearest(peaks(c, img))}}{|\boldsymbol{cells(img)}|}$$

$$distanceNearest(imgSeq) = \frac{\sum_{img:img \in imgs(imgSeq)} distanceNearest(img)}{|imgs(imgSeq)|}$$

- intensity ratio ($intensityRatio$), calculated as the ratio between intra-cell and inter-cell areas

$$intensityRatio(img) = \frac{\frac{\sum_{c:c \in cells(img)} cellBulkIntensity(c, img)}{|cells(img)|}}{backgroundIntensity(img)}$$

$$intensityRatio(imgSeq) = \frac{\sum_{img:img \in imgs(imgSeq)} cellBulkIntensity(img)}{|imgs(imgSeq)|}$$

Machine learning Methods:

All classification tasks were performed using the implementation in the HeuristicLab framework[50]. The following classification algorithms and parametrizations were applied:

- Random forests (RFs[51,52]): RFs are ensembles of decision trees, each depending on randomly chosen samples and features. Every tree votes for a certain label and the final assignment for the given sample is the mode vote of all trees (Parameters used: number of trees = 75).
- Support vector machines (SVMs[53]): SVMs are a widely used approach in machine learning based on statistical learning theory. The most important aspect of SVMs is the possibility to give bounds on the generalization error of the models produced, and to select the corresponding best model from a set of models following the principle of structural risk minimization. (We used polynomial kernels).
- Genetic programming (GP[49,54,55]): Genetic programming is an algorithmic concept that works with mathematical building blocks and iteratively compiles those to complex mathematical structures. As every evolutionary algorithm, GP works on populations of solution candidates and is based on selection, recombination, and mutation; here, we additionally use strict offspring selection. (Parameters used: mutation probability=15%, offspring selection, maximum tree length = 60).
- k-nearest neighbour algorithm (kNN[56]): k-nearest neighbour approaches work without creating and using any explicit models; a sample is classified using *k* training samples showing the smallest distance from the sample. (k was set to 5).

Data analysis – cell and single molecule detection

All data analysis tasks were performed using implemented and adapted image processing techniques. Details on the used image processing methods and the used parametrizations can be found in the Supplementary Material. First, we restricted the images (original image size 512x512 pixels) to a homogenously illuminated region of 300x300 pixels. All acquired images were further processed by applying the following image processing algorithms (using self-implemented C# algorithms and implementations in AForge.NET (http://www.aforgenet.com/)): Initially, images without any signal were discarded from further analyses by applying median thresholding. If less than three regions with higher intensity values than the median image intensity exist, the image will be discarded from further analyses and treated as a no-signal image[41]. This step is necessary in order to avoid false positive hits. All remaining images were further processed: First erythrocytes were detected in the corresponding bright field images. Cell detection was achieved by identification of the contours in the images. The position of the cell is estimated by assuming the erythrocyte cell position according to the identified contours, by optimizing this first cell position guesses, and finally, by optimizing the identified cell contours. Contour detection and identification were performed by applying a canny edge detector (low threshold=0, high threshold=3, Gaussian size=5, Gaussian sigma=1.4). Strongest contours were determined by applying a global threshold operation using a confidence threshold of 95%. The so created edge image is further processed by convoluting with a ring structured kernel (see Figure 2.b.I). As a result, first guess cell positions could be achieved. Those first guess cell positions were further optimised using an evolution strategy (mu=1, lambda=20, sigma=0.1, maximum number of iterations=100) resulting in a list of cell solution candidates. Those cell solution candidates were finally optimised in regard to optimal contours, by using an active contour method (alpha=2, beta=6, gamma=1.5, delta=1.0). Overlapping cells were discarded using an overlap threshold of 80% of the cell areas. In order to exclude malformed or chopped cells those with an area of less than 300 pixels were discarded[42,43,44,45]. Those methods allow detection of all erythrocytes in each image, regardless of their shape or size.

Subsequently, D antigen occurrences on the cell membrane were identified in the images. This step is crucial as all results depend on the correct detection of single

peaks. D antigen molecules were detected using the following algorithms and concepts: First, conservative smoothing with a kernel size of 5 was applied on the images in order to remove possible measurement artefacts. A top-hat filtering method with a self-defined structure element (as depicted in Figure 2.b.II) was used to detect sphere structures in the images. A global thresholding operation was applied (with a parameterised threshold of 15) to separate real signals from background signals. Finally, all possible signal regions were determined using an 8-connected region-growing algorithm. Regions that were smaller than a threshold of 5 were discarded[46,47].

Supplementary Note (including Supplementary Figures 8 and 9):

In order to further analyse the peak intensity signals on D- and DEL cells, we performed experiments with protein-G-coated glass slides. Protein G (Sigma Aldrich, Vienna, Austria) was adsorbed to the glass surface previously cleaned with piranha-solution. In two different experimental settings either fluorescently labelled anti-D antibody (Atto655-H41-Ab) or solely the fluorophore itself were attached to the coated surface. Protein G binds the Fc part of immunoglobulins and acts as a spacer to the glass surface. Thus, an interaction of the fluorophores or the fluorophore-labelled antibodies with the glass is avoided.

A statistical comparison of fluorescence signals of Atto655 and Atto655 marked anti-D antibodies with fluorescence signals on cells was performed as follows:

We applied a probability density fit algorithm, which estimates the average number of fluorophores per antibody, as originally described in [37,38] Briefly, this algorithm works as follows: Starting with the probability density P1(C) for counts C from a sample using a very high probability that each fluorescent spot represents only one fluorescent antibody, one extrapolates this probability density P1(C) to a probability density P2(C) for the hypothetical situation, where each spot contains exactly two antibodies by performing the convolution

$$P2(C) = \int P1(C') \, P1(C - C') \, dC' \qquad (1)$$

and then iterates the process for probability densities Pn(C) where each spot contains exactly n fluorophores

$$Pn(C) = \int P1(C') \, Pn\text{-}1\,(C - C') \, dC' \qquad (2)$$

In the next step, one compares the weighted sum of all of these probability densities with the empirical probability density Pemp(C) of a sample where the number of antibodies per fluorescing spot is unknown.

$$Pemp\,(C) \approx \sum_n w_n \, Pn\,(C) \qquad (3)$$

The analysis of the signals of mere Atto655 and Atto655-H41 antibodies (Supplementary Figure 8) shows that app. 87% of all Atto655-H41-Ab signals correspond to the signal of one fluorophore and the rest corresponds to the signal of more than one. Figure 8 displays the analysed populations; the black curve represents the Atto655-only signal, magenta the Atto655-H41-Ab distribution, and the blue the fitted convoluted signal [37,38].

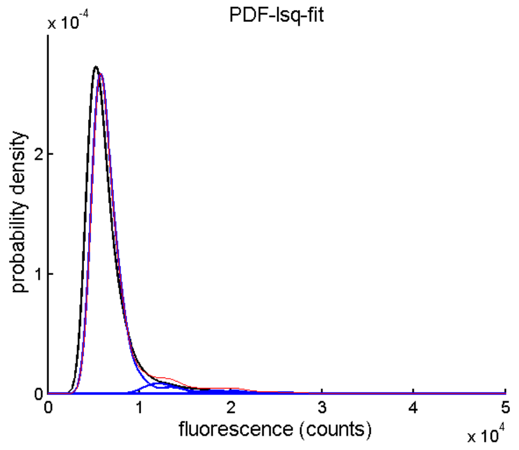

Supplementary Figure 8. Probability density distribution of Atto655-only (black line) and of Atto655-labelled H41 antibody on protein-G-coated glass slide (magenta line). The population of the fluorophores-only measurement is used for determination of distribution of Atto655-H41-Ab (fit shown as blue line). The maxima correspond to the one-, two-, three-, and fourfold of the average fluorescence intensity of single fluorophores. The signal of app. 87% of all detected Atto655-H41-Ab antibodies corresponds to the average fluorescence intensity of a single Atto655 fluorophore, the rest of the Atto655-H41-Ab signals corresponds to multiple Atto655 fluorophore signals.

We have compared the fluorescence signals of the Atto655-H41-Ab measured on protein-G-coated surface to the population of the Atto655-H41-Ab signals determined on the apical side of the D- cells. The statistical comparison (Figure 9A) shows that the distribution of the peak intensity signals of all cells overlap by app. 96%. This indicates that the majority of the peaks on the cells correspond to individual Atto655-H41-Ab signals; the rest corresponds to multiple fluorophores. Moreover, we statistically compared the signals determined on the apical side of DEL cells to the signals of Atto655-H41-Ab measured on protein-G-coated surface. The statistical analysis shows that the Atto655-H41-Ab signals determined from DEL population show >80% similarity (Figure 9.B.).

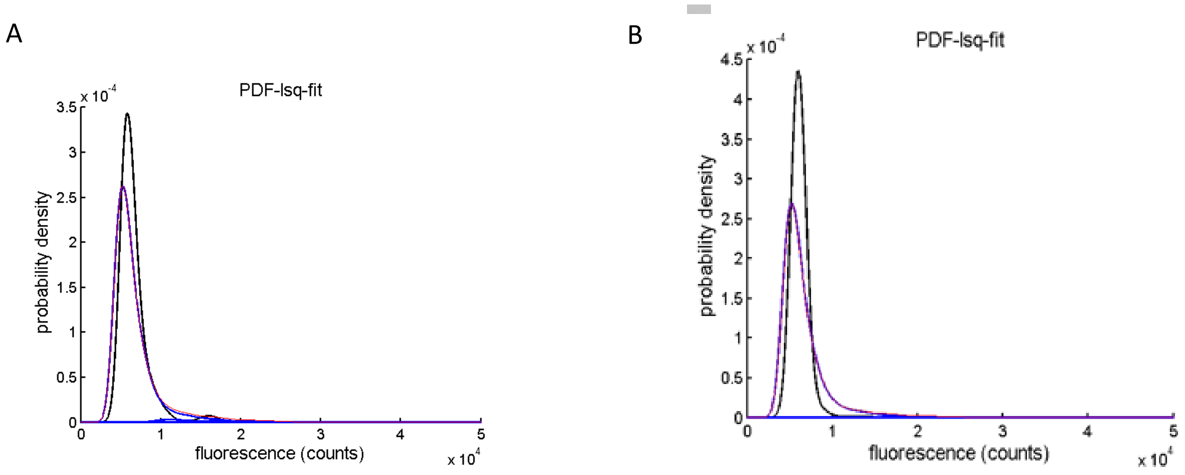

Figure 9.A. Probability density distribution of fluorescence intensity of Atto655-labelled H41 antibodies on a protein-G-coated glass slide (black line) and Atto655-H41 –Ab marked D- cells (magenta line). The Atto655-H41-Ab population is used for the determination of the population distribution on D- cells (blue). The maxima correspond to the one-, two-, three- and four-fold of the average fluorescence intensity of single Atto655-H41 antibodies labelled with a single fluorophore. App. 96% of all peak intensity signals correspond to the signal of single Atto655 labelled H41 antibodies. **9.B. Probability density distribution of Atto655-labelled H41 antibodies on protein-G-coated glass slides (black line) and the peak intensity distribution of Atto655-H41–Ab labelled DEL cells (magenta line).** The population of the Atto655-H41-Ab is used for the determination of the population distribution on DEL cells (fit shown as blue line). The maxima correspond to the one-, two-, three- and fourfold of the average fluorescence intensity of single Atto655-H41 antibodies labelled with a single fluorophore. The distributions overlap by >80% indicating that the majority of all peak intensity signals correspond to the signal of single Atto655 labelled H41 antibodies.

We would like to point out that the comparison of the fluorescence intensities is an estimation for the number of antibodies. Therefore, a value for the number of dimers and higher multimers by the homo-convolution of the monomer distribution provides only rough information about the number of antibodies that bind the RhD antigen. Our statistical analysis shows that the majority of all Atto655-H41-Ab signals correspond to the signal of individual Atto655 fluorophores, 13% of the signals have a higher intensity. Clustering of labelled and unlabelled antibodies cannot be excluded. Due to monoclonal antibody design, we do not expect a multiple binding of the antibodies to the RhD antigen (provided that the antigen carries just one epitope for the antibody as in our case). For cells with a low RhD expression (D- and DEL) a clustering of RhD via crosslinking of the antibodies is not highly probable. An intrinsic clustering of RhD proteins is not probable and not supported by any publications. We

expect that the antibodies have similar aggregation behaviour on all cells (in case of D- and DEL sparsely distributed antibodies) and protein-G-coated surfaces. This is supported by the statistical comparison between the populations of the Atto655-H41-Ab measured on protein-G-coated surface and the population of the Atto655-H41-Ab determined on the apical side of the D- cells (App. 96% of the population is statistically equal).

Hence, we show that for sparsely distributed RhD proteins on D- and DEL cells over 80% of the fluorescence signal distribution is similar to the distribution of single Atto655-labelled antibodies.
